# Supplementary material for: Obesity-associated microbiomes instigate visceral adipose tissue inflammation by recruitment of distinct neutrophils
Source: Nat Commun. 2024 Jun 27;15:5434. doi: 10.1038/s41467-024-48935-5 (PMC11211470; doi:10.1038/s41467-024-48935-5)
Supplement: Supplementary file 1 — Supplementary information [file 41467_2024_48935_MOESM1_ESM.pdf]

Supplementary Information

Obesity-associated microbiomes instigate visceral adipose tissue inflammation by recruitment of distinct neutrophils

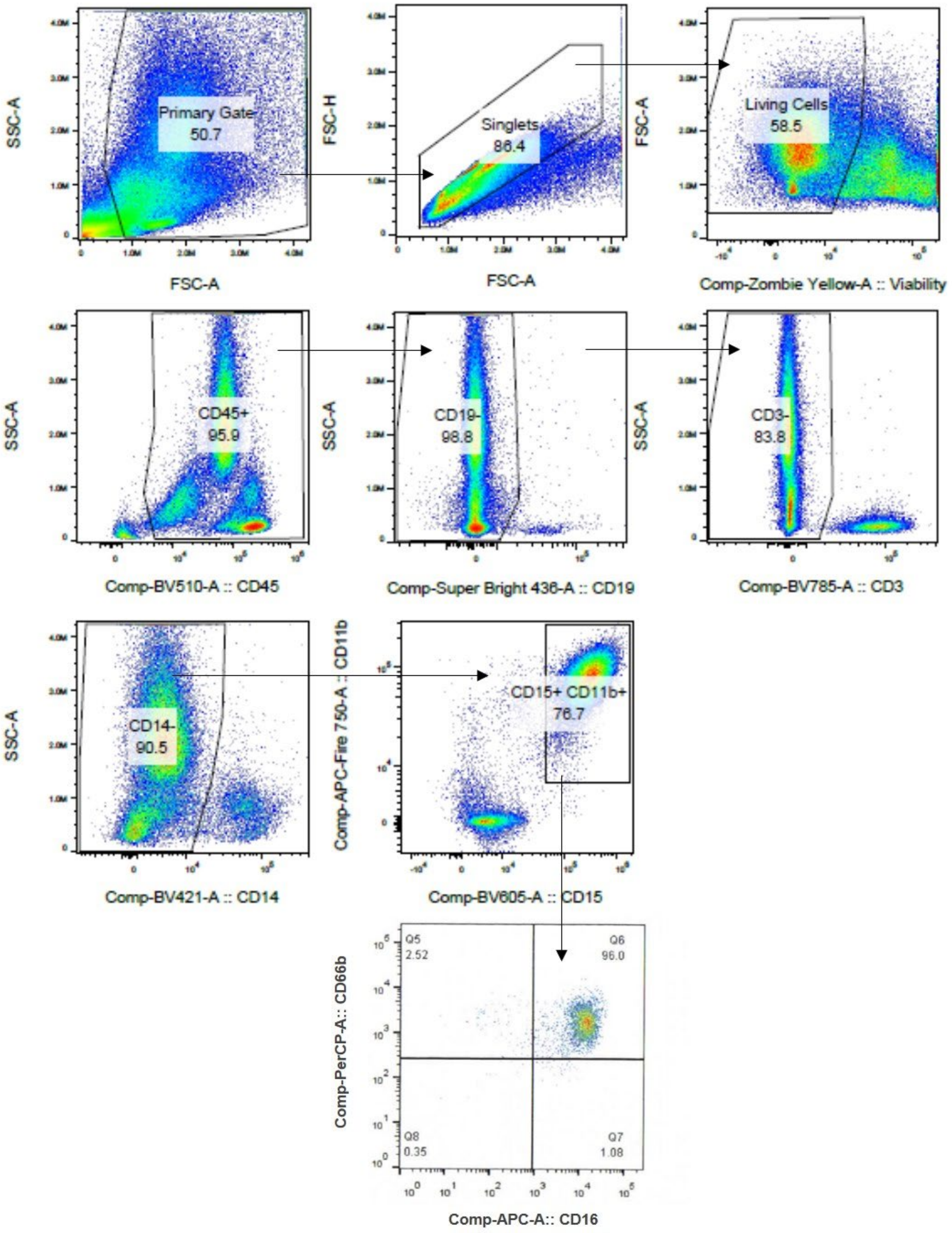

Supplementary figure 1. Representative flow gating scheme for human visceral adipose tissue (VAT) neutrophils, flow cytometry done on BD LSR II with data analyzed by FlowJo v10.8.1 software.

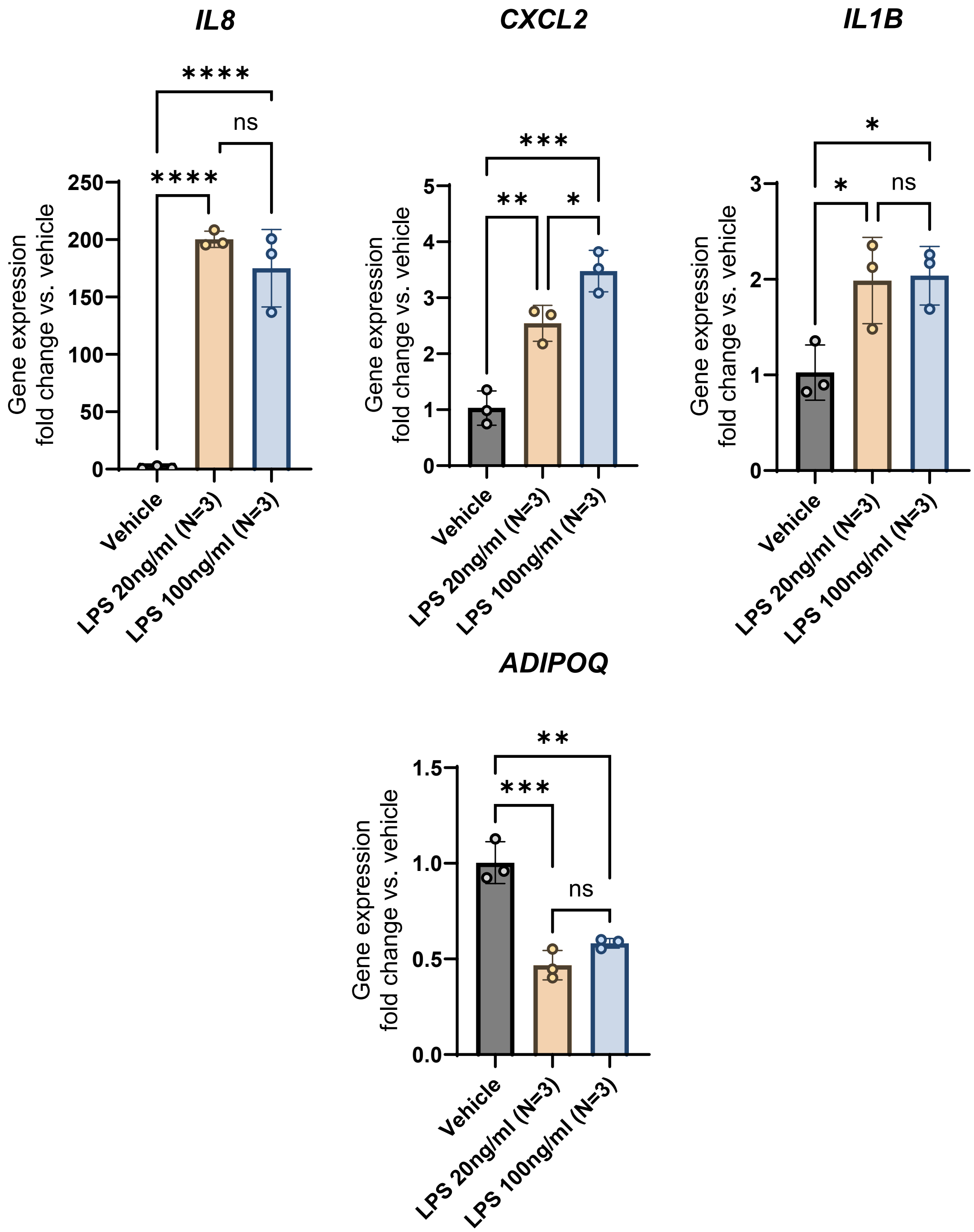

Supplementary figure 2. Gene expression analyses by qRT-PCR of cultured human adipocytes treated with LPS revealed an increase in *IL8, CXCL2, IL1B and* decrease in *ADIPOQ* gene expression (n=3), similar to differences between expression of these genes in adipocytes from humans with obesity vs. lean human adipocytes. All data represented as mean± SD compared by one-way ANOVA followed by post-hoc Tukey's multiple comparisons test with two-tailed analysis . t:p<0.1 *:p<0.05, **: p<0.01, ***: p<0.001, ****: p<0.0001

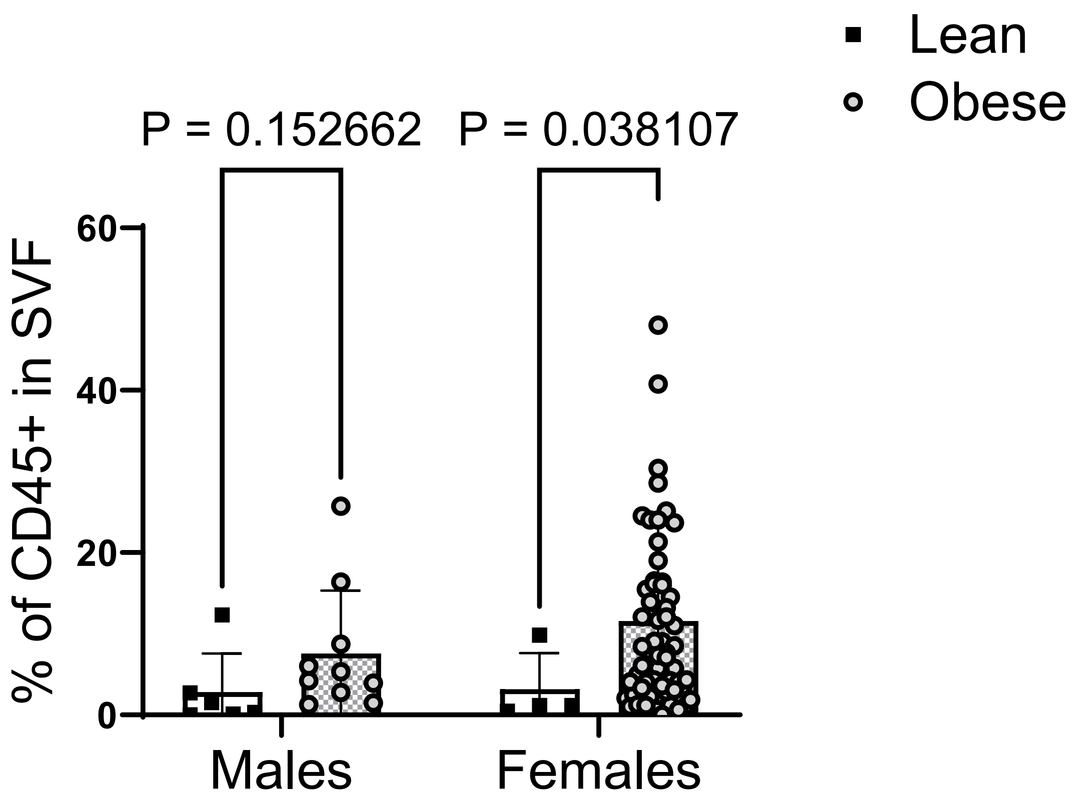

Supplementary figure 3. Effect of gender on % VAT neutrophil rise in obesity. % VAT neutrophils in subjects with obese (10 males and 63 females) and lean subjects (6 males and 4 females) measured with flow cytometry analysis. Data represented as mean± SD compared using unpaired student's t test with two-tailed analysis .

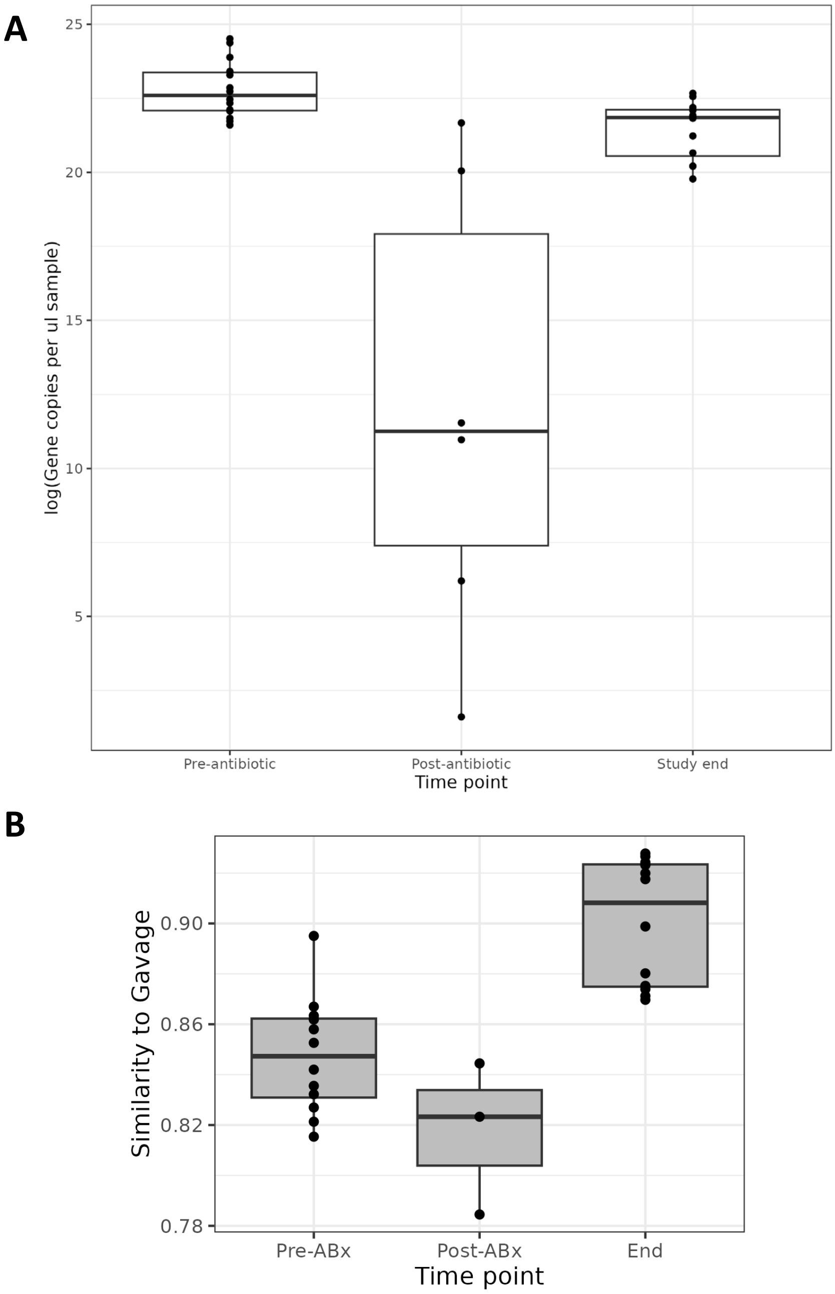

Supplementary Figure 4. Microbiome depletion and gavage efficacy. (A) Stool samples from mice after treatment with antibiotics showed fewer total bacteria than samples taken pre-antibiotics (p=0.0007206, Kruskal-Wallis) or at the end of the experiment (p=0.004958, Kruskal-Wallis), after gavage with human fecal sample (n=27). (B) The 16S community was more similar to the gavaged sample at the end of the study than either before the antibiotics (p=0.0001096, Kruskal-Wallis) or after the antibiotics, pre-gavage (p=0.009375, Kruskal-Wallis), as measured by inverse Gower's distance (n=27). Boxplots are defined using the first and third quartiles to bound the box. The minima is the first quartile less 1.5(inter-quartile range) and the maxima is the third quartile plus 1.5(inter-quartile range). Outliers are shown as points.

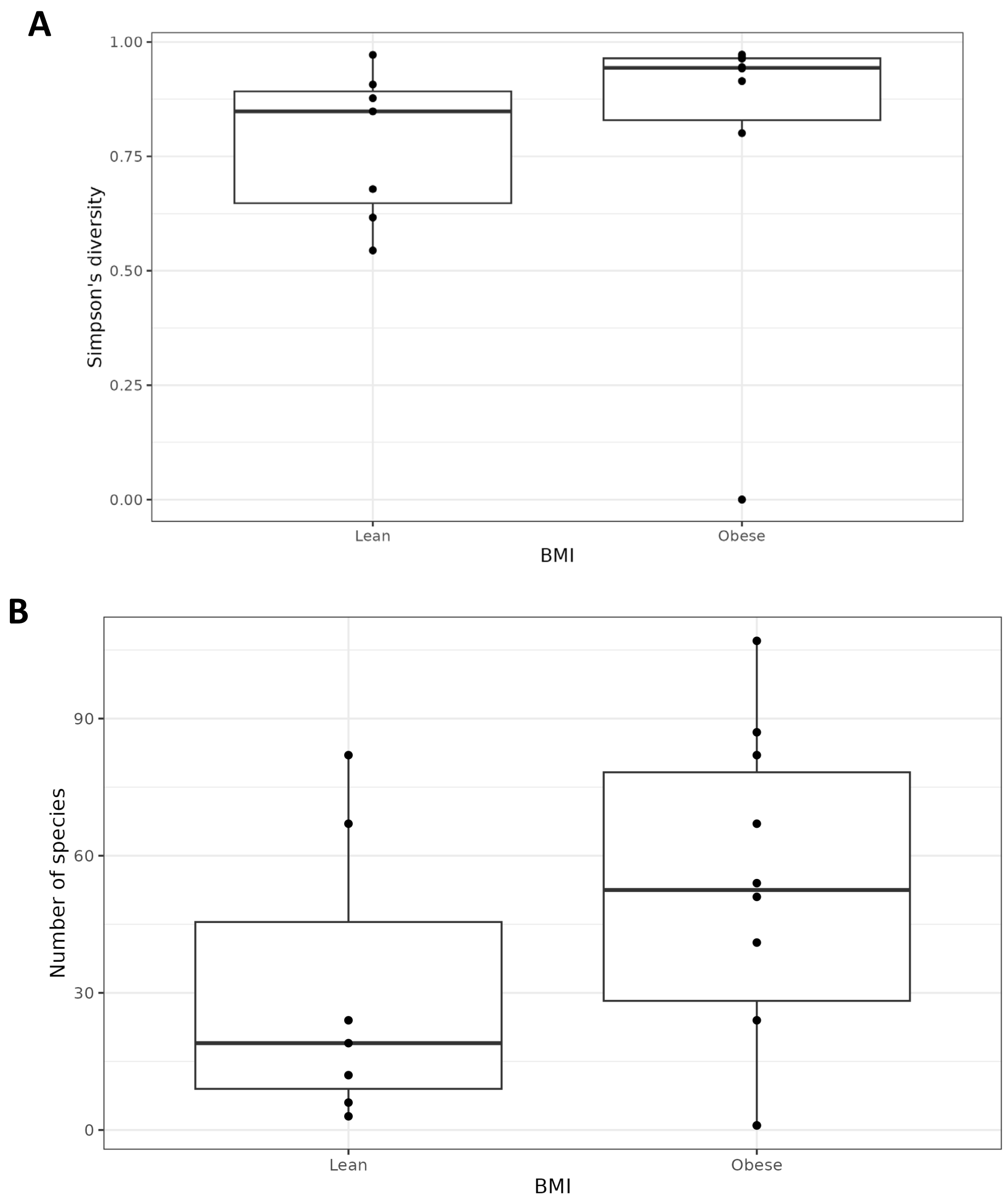

Supplementary Figure 5. Characteristics of the microbes in human VAT in lean subjects and subjects with obesity. The Simpson's diversity (A) and number of species observed (B) are not significantly different in the human VAT samples (n=17); (Kruskall-Wallace tests, p.value=0.2828 for diversity, p.value=0.3043 for species richness). Boxplots are defined using the first and third quartiles to bound the box. The minima is the first quartile less 1.5(inter-quartile range) and the maxima is the third quartile plus 1.5(inter-quartile range). Outliers are shown as points.

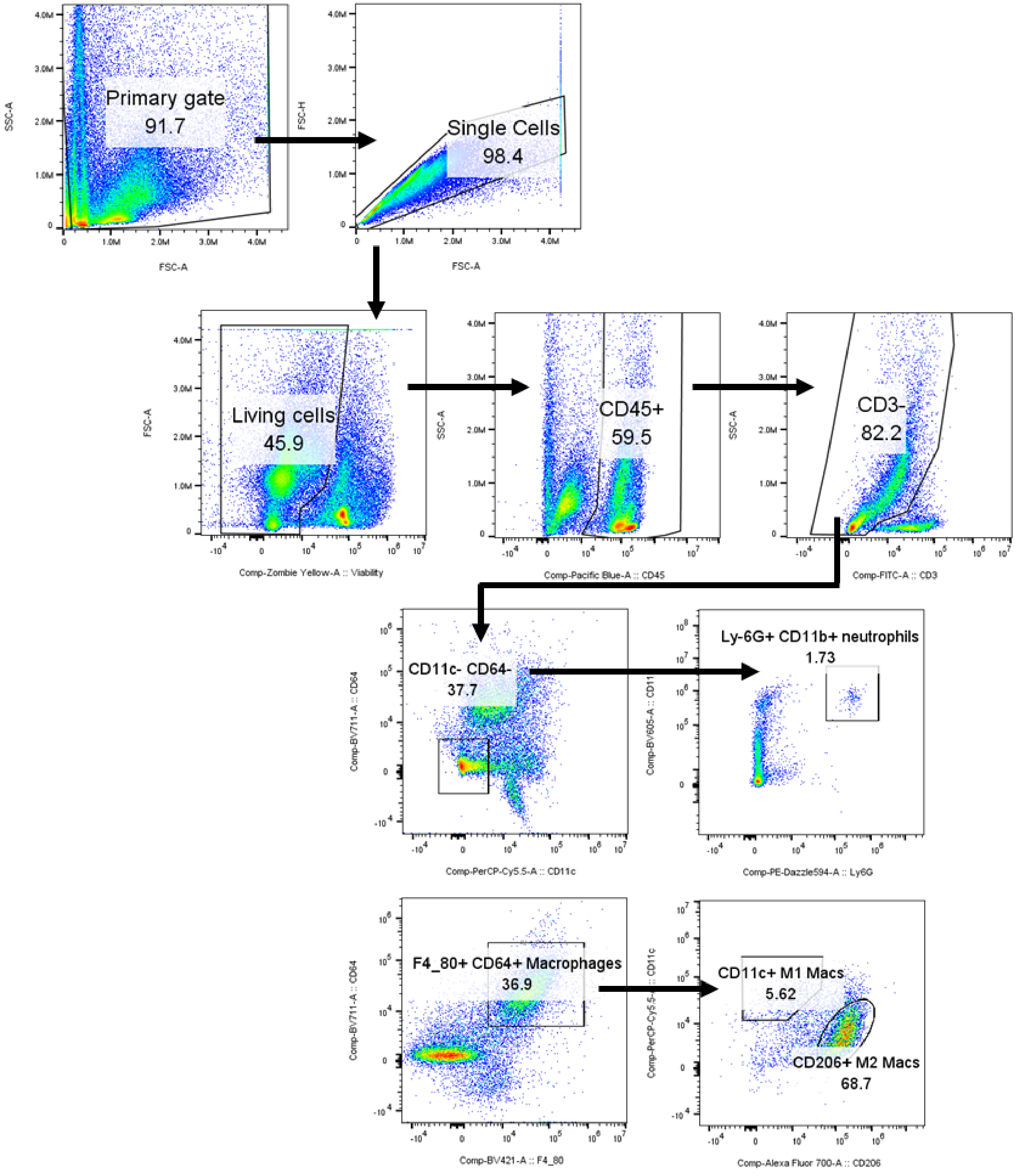

Supplementary Figure 6. Representative flow gating scheme for mouse VAT and splenic neutrophils and macrophages, flow cytometry done on cytek aurora and data analyzed with FlowJo v10.8.1 software.

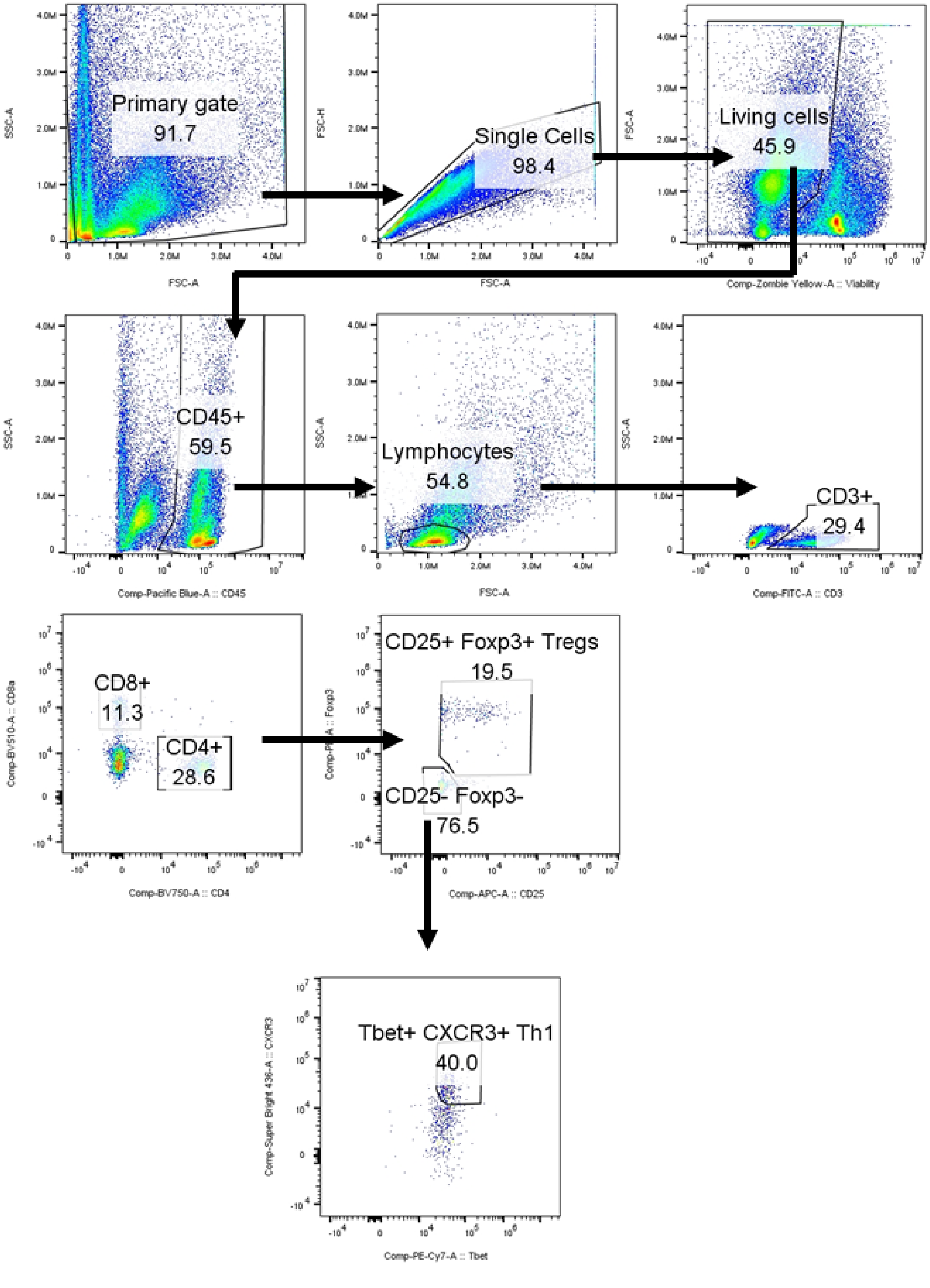

Supplemental Figure 7. Representative flow gating scheme for mouse VAT and splenic CD4, CD8, regulatory T cells (Tregs), T helper 1 (Th1) cells, flow cytometry done on cytek aurora and data analyzed with FlowJo v10.8.1 software.

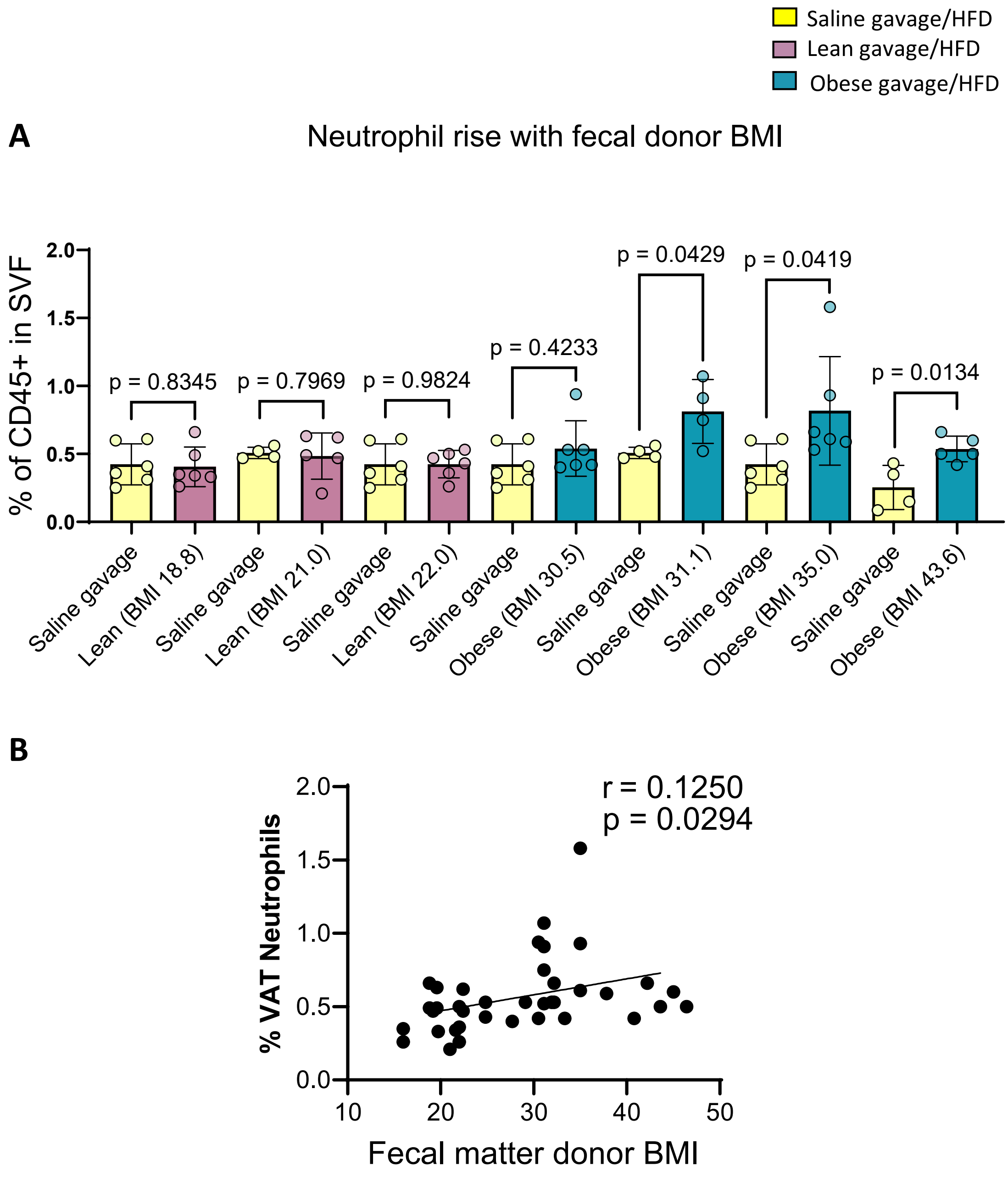

Supplementary figure 8. Rise in VAT neutrophil abundance corelates with the obesity status of the fecal gavage donors. A) % VAT neutrophils assessed by flow cytometry in the recipient mice (n=6 per group) that received obese fecal gavage from 3 lean individuals and 4 individuals with obesity compared to saline gavage, Data represented as mean± SD compared using multiple unpaired student's t test with two-tailed analysis. B) Correlation (Pearson) plot between fecal donor body mass index (BMI) and neutrophil abundance in adipose tissue of the recipient mice.

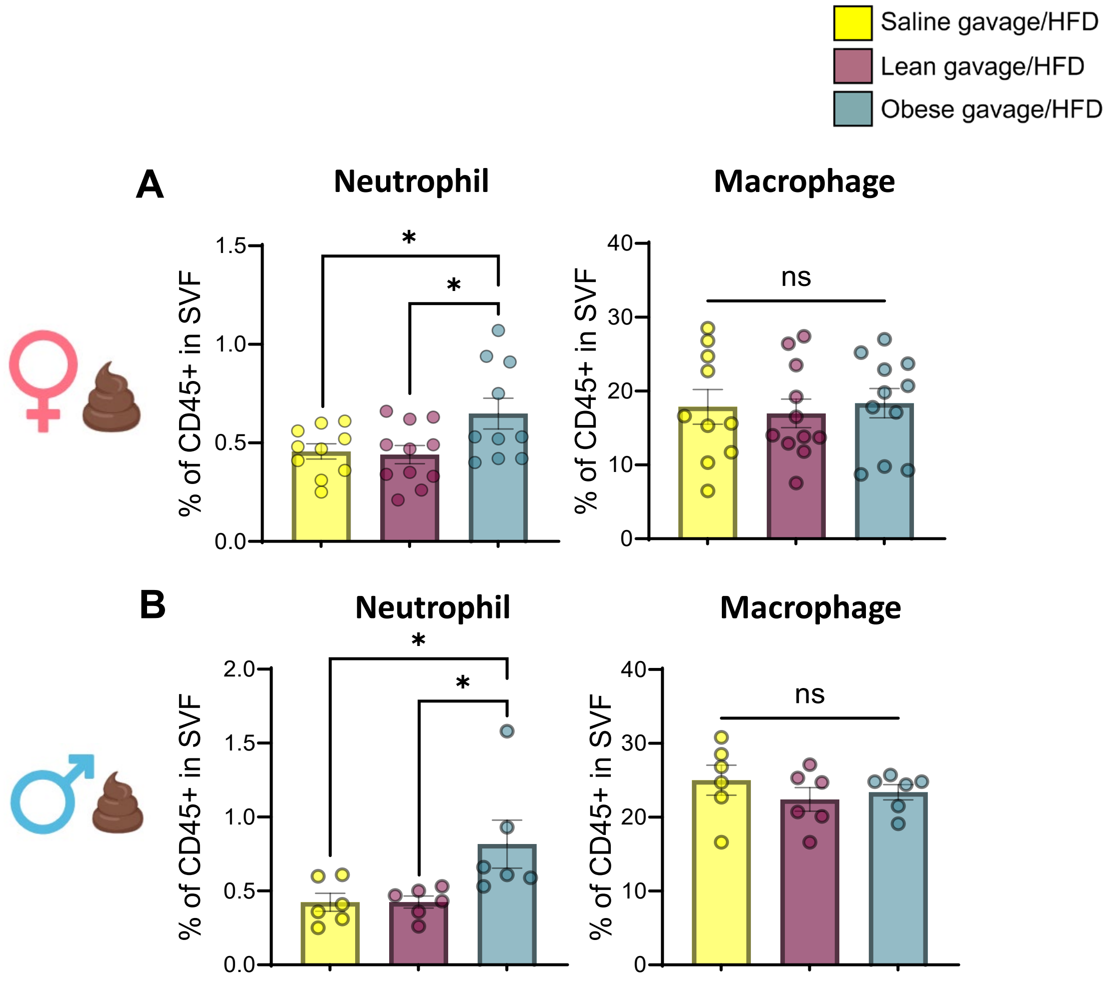

Supplementary figure 9. Human microbiome from both male and female fecal material drives the recruitment of neutrophils into adipose tissue (AT) in avatar mice. Stool samples from 3 individuals with obesity (1 male and 2 females) vs. 3 lean (1 male and 2 females) individuals and sterile saline control was gavaged into mice (n=6) per fecal sample). A) Gavaged stool from two obese females resulted in increased AT neutrophils in the adipose tissue compared to two lean females and saline gavage. No change in macrophages was observed. B) Consistently, stool gavage from a male with obesity also increased AT neutrophils but not macrophages compared to stool from one lean male and a saline control. All flow cytometry data represented as mean± SD compared by one-way ANOVA followed by post-hoc Tukey's multiple comparisons test with two-tailed analysis . *:p <0.05. (Figure components created with BioRender.com released under a Creative Commons Attribution-NonCommercial-NoDerivs 4.0 International license https://creativecommons.org/licenses/by-nc-nd/4.0/deed.en).

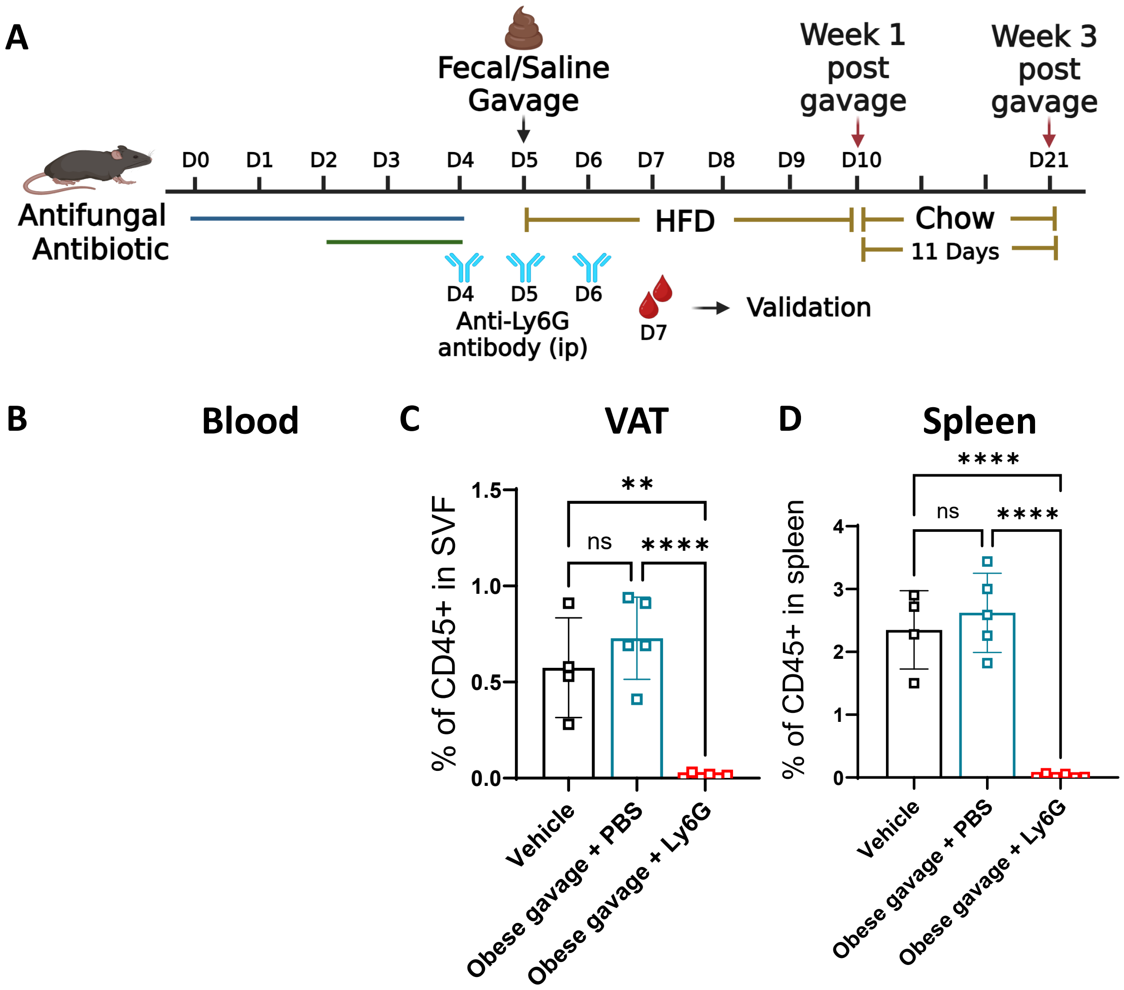

Supplementary Figure 10. A) Experimental design and validation of neutrophil depletion by αLy6G antibody. (Image created using BioRender). Flow cytometry analysis on submandibular blood collected post 3 αLy6G injections confirmed the depletion of neutrophils in B) blood, C) VAT and D) Spleen (n=6/ group). Flow analysis on VAT and spleen was performed at the time of sacrifice at weeks 1 and 3 as detailed in the protocol. All data represented as mean± SD compared by one-way ANOVA followed by post-hoc Tukey's multiple comparisons test with two-tailed analysis-. *:$p<0.05$, **: $p<0.01$, ***: $p<0.001$, ****: $p<0.0001$. (Figure A, created with BioRender.com released under a Creative Commons Attribution-NonCommercial-NoDerivs 4.0 International license https://creativecommons.org/licenses/by-nc-nd/4.0/deed.en).created with BioRender.com)

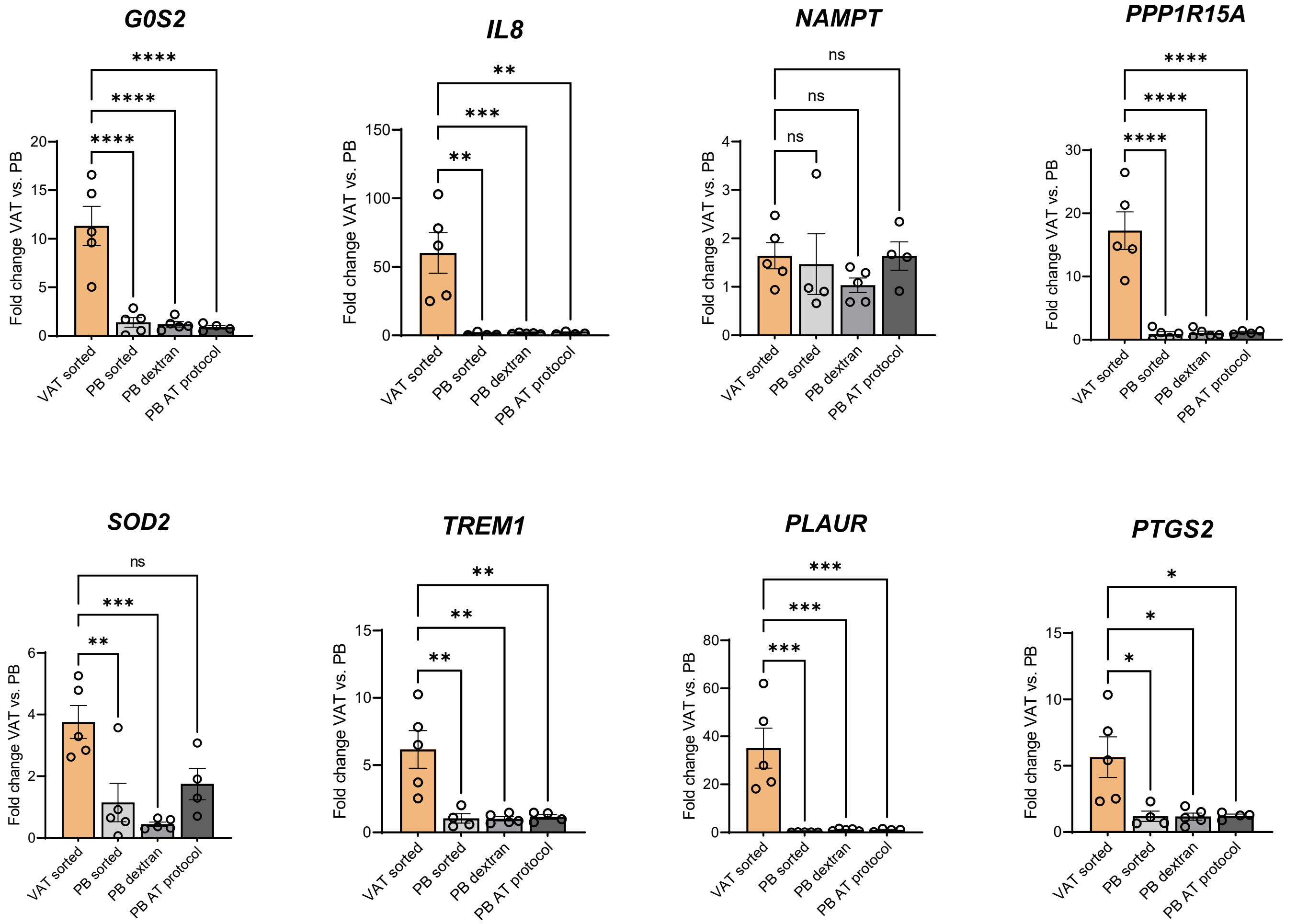

Supplementary Figure 11. qRT-PCR validation of VAT neutrophil signature genes compared to peripheral blood (PB) neutrophils (n=5). In order to test the robustness of the VAT neutrophil signature validation, PB neutrophils were isolated by flow sorting, the commonly used dextran method, and subjected to VAT neutrophils isolation conditions (i.e. dissociation buffers, temperatures etc.). All conditions showed similar levels of gene expression. All data represented as mean± SD compared by one-way ANOVA followed by post-hoc Tukey's multiple comparisons test with two-tailed analysis. *:p<0.05, **: p<0.01, ***: p<0.001, ****: p<0.0001

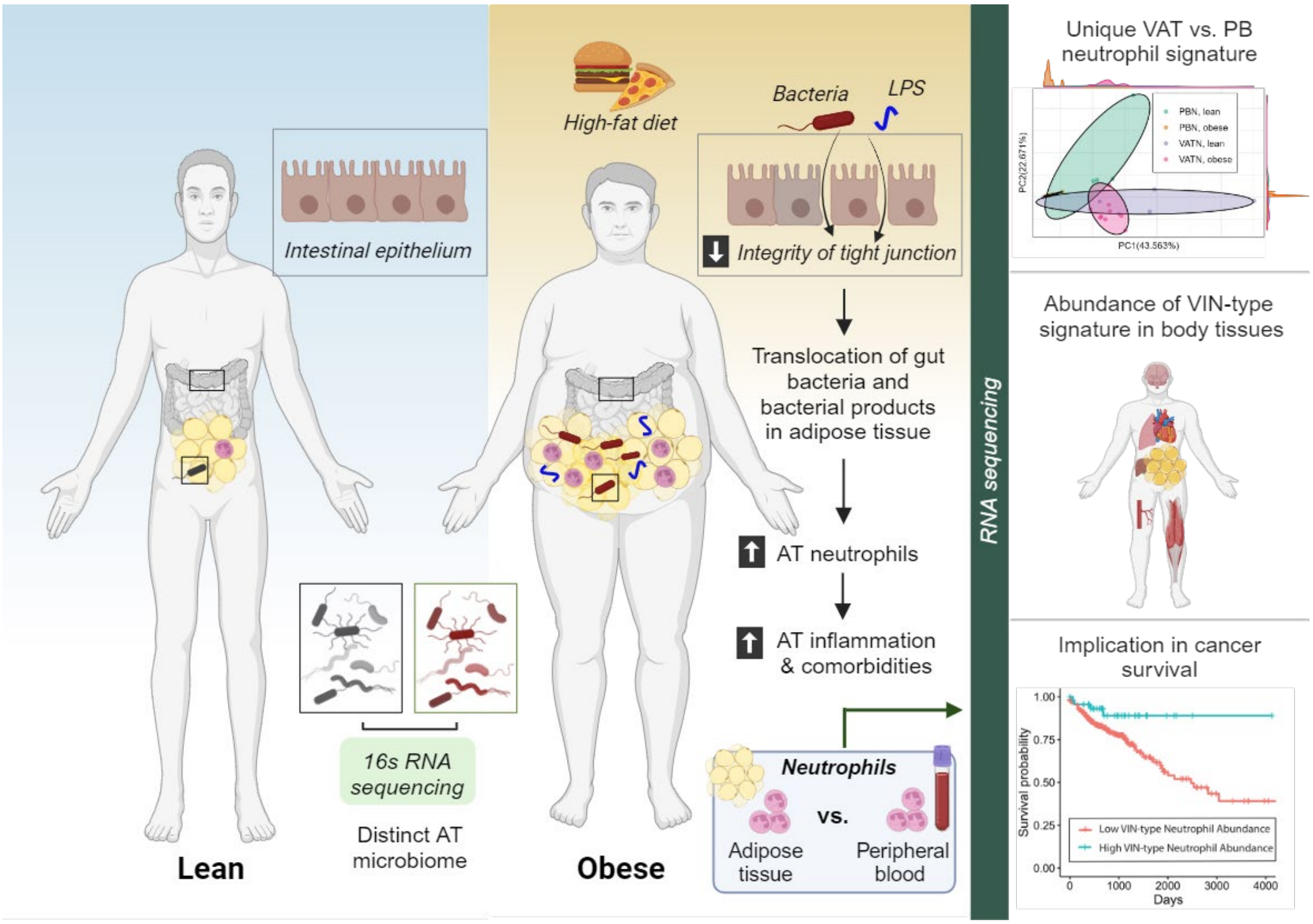

Image created using Biorender.com

Supplementary Figure 12. Summary of the proposed concept illustrating the role of obesity-associated microbiomes in instigating visceral adipose tissue inflammation by recruitment of distinct neutrophils. (Figure created with BioRender.com released under a Creative Commons Attribution-NonCommercial-NoDerivs 4.0 International license https://creativecommons.org/licenses/by-nc-nd/4.0/deed.en)

Donor ID	Age	Sex	BMI	Lean/ Obese	Hypertension/ Dyslipidemia/other	Medications
T6291	35-40	F	21	L	NO	Birth control
T6824	20-25	F	31.1	O	NO	NO
T5631	20-25	M	21.9	L	NO	NO
T6821	25-30	M	34.96	O	NO	NO
T6557	30-35	F	18.79	L	NO	Cetirizine, Nexplanon
T6820	25-30	F	30.45	O	NO	NO
T7154	30-35	F	43.6	O	NO	NO

Supplementary table 1. Demographics of the fecal sample donors.

Neutrophil Group Label	accession	Tissue Source	control	treatment
Airspace	GSE2322	alveolar space, circulating	circulating	alveolar space
Active.TB	GSE19443	blood	healthy	Active.TB
Endotox	GSE2322	blood	untreated	endotoxin
Sepsis	GSE64457	blood	Healthy control	Patient
Exercise	GSE8668	blood	before exercise	after exercise
Synovial	GSE116899	blood, synovial	blood	synovial
VAT	This study	blood, VAT	blood	VAT
Lung.Cancer	GSE68795	lung	normal tissue	tumor

Supplementary table 2. Accession numbers and sources of isolated neutrophils compared to those from visceral adipose tissue (VAT) and peripheral blood (PB).

	Subjects (n=6)
**BMI (kg/m²)**	39.4 ± 4.4
**Age (years)**	44 ± 4.6
**Gender**	4F/2M
**Presence of diabetes**	3 out of 6
**Fasting Glucose (mg/dL)**	96 ± 8.4
**Total Cholesterol (mg/dL)**	160 ± 12.7
**Triglycerides (mg/dL)**	115 ± 14.5
**LDL**	88 ± 13.5
**HDL**	49 ± 2.8
**ALT**	24 ± 2.4
**AST**	25 ± 3.3

Supplementary table 3: Patient demographics for the gene expression validation cohort with all values expressed as mean +/- SEM.

Accession	Tissue
GSE103889	Blood
GSE14771	Blood
GSE28064	Blood
GSE19790	Blood
GDS4276	VAT
GSE71415	VAT

Supplementary table 4. Accession numbers and sources of transcriptome data used to validate the custom visceral adipose tissue (VAT) neutrophil signature

	Lean (n=6)	Obese (n=9)
**BMI (kg/m^2)**	23.2 ± 0.5	36.5 ± 2.4
**Age (years)**	58 ± 6.1	50.4 ± 3.3
**Gender**	2F/4M	8F/1M

Supplementary table 5. Patient demographics for the transcriptome analysis using ampliseq analysis between VAT vs. PB neutrophils in lean individuals (n=6) and individuals with obesity (n=9). All values expressed as mean +/- SEM.

Species	Gene	Forward Primer (5' - 3')	Reverse Primer (5' - 3')	Company	Catalogue #
Hu	*ADIPOQ*	GGTCTTATTGGTCCTAAGGG	GTAGAAGATCTTGGTAAAGCG	Sigma	KSPQ12012-9370
Hu	*IL1B*	CTAAACAGATGAAGTGCTCC	GGTCATTCTCCTGGAAGG	Sigma	KSPQ12012-3553
Hu	*NLRP3*	AGGTGTTGGAATTAGACAAC	AATACATTTCAGACAACCCC	Sigma	KSPQ12012-114548
Hu	*TNF*	AGGCAGTCAGATCATCTTC	TTATCTCTCAGCTCCACG	Sigma	KSPQ12012-50486
Hu	*LEP*	TCAATGACATTTCACACACG	TCCATCTTGGATAAGGTCAG	Sigma	KSPQ12012-3952
Hu	*IL8*	GTTTTTGAAGAGGGCTGAG	TTTGCTTGAAGTTTCACTGG	Sigma	KSPQ12012-3576
Hu	*G0S2*	GCCACTAAGGTCATTCCCG	TTCACCATCTTCCCCTTGC	Sigma	KSPQ12012-50486
Hu	*SOD2*	ATCATACCCTAATGATCCCAG	AGGACCTTATAGGGTTTTCAG	Sigma	KSPQ12012-6648
Hu	*NAMPT*	CTAATGGCCTTGGGATTAAC	TCCAGTGTAACAAAATTCCC	Sigma	Oligo synthsis
Hu	*PPP1R15A*	GAAACCCCTACTCATGATCCG	AAATGGACAGTGACCTTCTCG	Sigma	Oligo synthsis
Hu	*TREM1*	GGAAGGATGAGGAAGACCAG	TCACATTTCACATCCAGGGTC	Sigma	Oligo synthsis
Hu	*PLAUR*	ACAACGACACCTTCCACTTC	GGCAGATTTTCAAGCTCCAG	Sigma	Oligo synthsis
Hu	*PTGS2*	ACAGGCTTCCATTGACCAG	TCACCATAGAGTGCTTCCAAC	Sigma	Oligo synthsis
Hu	*PPIA*	GGCAAATGCTGGACCCAACACA	TGCTGGTCTTGCCATTCCTGGA	Sigma	KSPQ12012-5478
Ms	*Ly6g*	CCCTTCTCTGATGGATTTTG	AGTATTGTCCAGAGTAGTGG	Sigma	KSPQ12012-546644
Ms	*Emr1*	TTTCAAATGGATCCAGAAGG	CAGAAGGAAGCATAACCAAG	Sigma	KSPQ12012-13733
Ms	*Il1b*	GGATGATGATGATAACCTGC	CATGGAGAATATCACTTGTTGG	Sigma	KSPQ12012-16176
Ms	*Spp1*	GGATGAATCTGACGAATCTC	GCATCAGGATACTGTTCATC	Sigma	KSPQ12012-20750
Ms	*Ppia*	TTCACCTTCCCAAAGACCAC	CAAACACAAACGGTTCCCAG	IDT	185069231, -232

Supplementary table 6. Sequences of oligonucleotides for primers used for qRT-PCR.
