# Supplementary material for: Obesity-associated microbiomes instigate visceral adipose tissue inflammation by recruitment of distinct neutrophils
Source: Nat Commun. 2024 Jun 27;15:5434. doi: 10.1038/s41467-024-48935-5 (PMC11211470; doi:10.1038/s41467-024-48935-5)
Supplement: Supplementary file 3 — Reporting Summary [file 41467_2024_48935_MOESM3_ESM.pdf]

nature portfolio

Corresponding author(s): Dr. Willa Hsueh

Last updated by author(s): 2024-04-08

Reporting Summary

Nature Portfolio wishes to improve the reproducibility of the work that we publish. This form provides structure for consistency and transparency in reporting. For further information on Nature Portfolio policies, see our Editorial Policies and the Editorial Policy Checklist.

Please do not complete any field with "not applicable" or n/a. Refer to the help text for what text to use if an item is not relevant to your study.
For final submission: please carefully check your responses for accuracy; you will not be able to make changes later.

Statistics

For all statistical analyses, confirm that the following items are present in the figure legend, table legend, main text, or Methods section.

n/a	Confirmed	
☐	☑	The exact sample size (*n*) for each experimental group/condition, given as a discrete number and unit of measurement
☐	☑	A statement on whether measurements were taken from distinct samples or whether the same sample was measured repeatedly
☐	☑	The statistical test(s) used AND whether they are one- or two-sided<br>*Only common tests should be described solely by name; describe more complex techniques in the Methods section.*
☐	☑	A description of all covariates tested
☐	☑	A description of any assumptions or corrections, such as tests of normality and adjustment for multiple comparisons
☐	☑	A full description of the statistical parameters including central tendency (e.g. means) or other basic estimates (e.g. regression coefficient) AND variation (e.g. standard deviation) or associated estimates of uncertainty (e.g. confidence intervals)
☐	☑	For null hypothesis testing, the test statistic (e.g. *F*, *t*, *r*) with confidence intervals, effect sizes, degrees of freedom and *P* value noted<br>*Give P values as exact values whenever suitable.*
☑	☐	For Bayesian analysis, information on the choice of priors and Markov chain Monte Carlo settings
☐	☑	For hierarchical and complex designs, identification of the appropriate level for tests and full reporting of outcomes
☐	☑	Estimates of effect sizes (e.g. Cohen's *d*, Pearson's *r*), indicating how they were calculated

Our web collection on statistics for biologists contains articles on many of the points above.

Software and code

Policy information about availability of computer code

Data collection	R analyses were run using Rv4.1.0, with some initial processing in Rv4.0.2. R packages used in the accesss and analysis of data include dada2 v1.12.1, tidyverse v2.0.0, ggdendro v0.1.23, vegan v2.5.7, broom v1.0.4, readr v2.1.4, survival v3.3.1, survminer v0.4.9, tmesig v0.1.0, RColorBrewer v1.1.3, viridis 0.6.5, ggrepel v0.9.1, ggfortify v0.4.14, ggforce v0.3.3, flextable v0.7.2, Seurat v4.4.0, decontam v1.4.0, HMP16SData v1.8.2, MGnifyR v0.1.0, and GenomicDataCommons v1.15.0. In addition, the tools sourcetracker v2.0.1, the CIBERSORT website (now available as CIBERSORTx[https://cibersortx.stanford.edu/]), FlowJo v10.8.1, and GraphPad Prism 10, were used. Code to regenerate all figures and analyses is available at
Data analysis	R analyses were run using Rv4.1.0, with some initial processing in Rv4.0.2. R

For manuscripts utilizing custom algorithms or software that are central to the research but not yet described in published literature, software must be made available to editors and reviewers. We strongly encourage code deposition in a community repository (e.g. GitHub). See the Nature Portfolio guidelines for submitting code & software for further information.

Data

Policy information about availability of data

All manuscripts must include a data availability statement. This statement should provide the following information, where applicable:

- Accession codes, unique identifiers, or web links for publicly available datasets
- A description of any restrictions on data availability
- For clinical datasets or third party data, please ensure that the statement adheres to our policy

The gene expression and microbiome sequencing data generated for this manuscipt are publicly available through the NCBI Bioproject ID: PRJNA766535 [https://www.ncbi.nlm.nih.gov/bioproject/PRJNA766535/]. Additional publicly available data from NCBI can be accessed at: GSE2322 [https://www.ncbi.nlm.nih.gov/geo/query/acc.cgi?acc=GSE2322], GSE19443 [https://www.ncbi.nlm.nih.gov/geo/query/acc.cgi?acc=GSE19443], GSE64457 [https://www.ncbi.nlm.nih.gov/geo/query/acc.cgi?acc=GSE64457], GSE8668 [https://www.ncbi.nlm.nih.gov/geo/query/acc.cgi?acc=GSE8668], [illegible]

Research involving human participants, their data, or biological material

Policy information about studies with human participants or human data. See also policy information about sex, gender (identity/presentation), and sexual orientation and race, ethnicity and racism.

Reporting on sex and gender	Described in the demographics table for all respective results, also any dditional details regarding human participants will be made available through the corresponding author of the manuscript, Willa.Hsueh@osumc.edu
Reporting on race, ethnicity, or other socially relevant groupings	Described in the demographics tables.
Population characteristics	Decribed in the demographics tables.
Recruitment	Participants were recruited as Lean (Body Mass Index (BMI=18-24) and Obese (BMI=30 and above) for the study. Participants were excluded if on any prescribed chronic steroid or anti-inflammatory agent, had end-stage renal or liver disease, or had a past diagnosis of Acquired Immune Deficiency Syndrome (AIDS) or neoplastic disease. No other selection bias was involved.
Ethics oversight	All human and mouse studies were approved by The Ohio State University Institutional Review Board (IRB # 2014H0471) and The Institutional Animal Care and Use Committee (IACUC# 2014A00000108-R3) respectively.

Note that full information on the approval of the study protocol must also be provided in the manuscript.

Field-specific reporting

Please select the one below that is the best fit for your research. If you are not sure, read the appropriate sections before making your selection.

☑ Life sciences ☐ Behavioural & social sciences ☐ Ecological, evolutionary & environmental sciences

For a reference copy of the document with all sections, see nature.com/documents/nr-reporting-summary-flat.pdf

Life sciences study design

All studies must disclose on these points even when the disclosure is negative.

Sample size	Sample size was determined based on similar studies in the field, sample availability and power analysis.
Data exclusions	All data outliers have been shown in the figures as is, and none have been excluded.
Replication	To ensure robust reproducibility, each experiment consisted of at least 3 biological replicates with minimum of 3 technical replicates. All attempts at replication were successful.
Randomization	This does not apply since all the mice allocated for experimental groups were ordered from Jackson labs (strain/ age/ diet details are given in the methods section) at an identical age and diet. However, we matched their body weights before starting the experimental procedure as weight can introduce certain bias. We made sure that the group of mice allocated to different treatmnets had similar body weight average as a group.
Blinding	No blinding was performed in mouse or human experiments. However, coading and decoding of the samples was done intermittently while repeating the same experiment to check the reproducibility of the results.

Behavioural & social sciences study design

All studies must disclose on these points even when the disclosure is negative.

Study description	
Research sample	
Sampling strategy	
Data collection	
Timing	
Data exclusions	
Non-participation	
Randomization	

Ecological, evolutionary & environmental sciences study design

All studies must disclose on these points even when the disclosure is negative.

Study description	
Research sample	
Sampling strategy	
Data collection	
Timing and spatial scale	
Data exclusions	
Reproducibility	
Randomization	
Blinding	

Did the study involve field work? ☐ Yes ☐ No

Field work, collection and transport

Field conditions	
Location	
Access & import/export	
Disturbance	

Reporting for specific materials, systems and methods

We require information from authors about some types of materials, experimental systems and methods used in many studies. Here, indicate whether each material, system or method listed is relevant to your study. If you are not sure if a list item applies to your research, read the appropriate section before selecting a response.

Materials & experimental systems

n/a	Involved in the study	
☐	☑	Antibodies
☑	☐	Eukaryotic cell lines
☑	☐	Palaeontology and archaeology
☐	☑	Animals and other organisms
☑	☐	Clinical data
☑	☐	Dual use research of concern
☑	☐	Plants

Methods

n/a	Involved in the study	
☑	☐	ChIP-seq
☐	☑	Flow cytometry
☑	☐	MRI-based neuroimaging

Antibodies

Antibodies used	Human neutrophils:LIN- (FITC: CD3 (Cat#:300440, Clone: UCHT1), CD14 (Cat#: 325604, Clone: HCD14), CD19 (Cat#: 302206, Clone: HIB19), CD20 (Cat#: 302304, Clone: 2H7), CD56 (Cat#: 318304, Clone: HCD56)), CD16 (APC, Cat#: 302012, Clone: 3G8), CD11b (BV421, Cat#: 301324, Clone: ICRF44), and CD15 (BV605, Cat#: 323032, Clone: W6D3), CD66b (PerCP-Cy5.5, Cat#: 305108, Clone: G10F5) and CD62L (PE, Cat#: DREG-56, Clone: 304805). Mouse neutrophils: Lin- CD3(Cat#100204, Clone:17A2) , CD19(Cat# 115506, Clone:6D5), NK1.1(Cat#108706, Clone:PK136), CD45+(Cat#103126, Clone:30-F11), CD11b+(Cat#101257, Clone:M1/70), Ly6G+(Cat#127648, Clone:1A8), CD64-
Validation	Human neutrophils:LIN- (FITC: CD3 (Cat#:300440, Clone: UCHT1), CD14 (Cat#: 325604, Clone: HCD14), CD19 (Cat#: 302206, Clone: HIB19), CD20 (Cat#: 302304, Clone: 2H7), CD56 (Cat#: 318304, Clone: HCD56)), CD16 (APC, Cat#: 302012, Clone: 3G8), CD11b (BV421, Cat#: 301324, Clone: ICRF44), and CD15 (BV605, Cat#: 323032, Clone: W6D3), CD66b (PerCP-Cy5.5, Cat#: 305108, Clone: G10F5) and CD62L (PE, Cat#: DREG-56, Clone: 304805). Mouse neutrophils: Lin- CD3(Cat#100204, Clone:17A2) , CD19(Cat# 115506, Clone:6D5), NK1.1(Cat#108706, Clone:PK136), CD45+(Cat#103126, Clone:30-F11), CD11b+(Cat#101257, Clone:M1/70), Ly6G+(Cat#127648, Clone:1A8), CD64-

Eukaryotic cell lines

Policy information about cell lines and Sex and Gender in Research

Cell line source(s)	
Authentication	
Mycoplasma contamination	
Commonly misidentified lines (See ICLAC register)	

Palaeontology and Archaeology

Specimen provenance	
Specimen deposition	
Dating methods	

☐ Tick this box to confirm that the raw and calibrated dates are available in the paper or in Supplementary Information.

Ethics oversight	

Note that full information on the approval of the study protocol must also be provided in the manuscript.

Animals and other research organisms

Policy information about studies involving animals; ARRIVE guidelines recommended for reporting animal research, and Sex and Gender in Research

Laboratory animals	Eight-week-old C57BL/6 male mice (Jackson lab; strain 000664) were used for
Wild animals	No wild animals were used. Only C57BL/6 male mice (Jackson lab; strain
Reporting on sex	All animal studies were performed in male mice.
Field-collected samples	No samples were collected in the field.
Ethics oversight	All the animal studies were approved by The Ohio State University Animal Care

Note that full information on the approval of the study protocol must also be provided in the manuscript.

Clinical data

Policy information about clinical studies

All manuscripts should comply with the ICMJE guidelines for publication of clinical research and a completed CONSORT checklist must be included with all submissions.

Clinical trial registration	
Study protocol	
Data collection	
Outcomes	

Dual use research of concern

Policy information about dual use research of concern

Hazards

Could the accidental, deliberate or reckless misuse of agents or technologies generated in the work, or the application of information presented in the manuscript, pose a threat to:

No	Yes	
☑	☐	Public health
☑	☐	National security
☑	☐	Crops and/or livestock
☑	☐	Ecosystems
☑	☐	Any other significant area

Experiments of concern

Does the work involve any of these experiments of concern:

No	Yes	
☑	☐	Demonstrate how to render a vaccine ineffective
☑	☐	Confer resistance to therapeutically useful antibiotics or antiviral agents
☑	☐	Enhance the virulence of a pathogen or render a nonpathogen virulent
☑	☐	Increase transmissibility of a pathogen
☑	☐	Alter the host range of a pathogen
☑	☐	Enable evasion of diagnostic/detection modalities
☑	☐	Enable the weaponization of a biological agent or toxin
☑	☐	Any other potentially harmful combination of experiments and agents

Plants

Seed stocks

Novel plant genotypes

Authentication

ChIP-seq

Data deposition

☐ Confirm that both raw and final processed data have been deposited in a public database such as GEO.

☐ Confirm that you have deposited or provided access to graph files (e.g. BED files) for the called peaks.

Data access links
May remain private before publication.

Files in database submission

Genome browser session
(e.g. UCSC)

Methodology

Replicates

Sequencing depth

Antibodies

Peak calling parameters

Data quality

Software

Flow Cytometry

Plots

Confirm that:

☑ The axis labels state the marker and fluorochrome used (e.g. CD4-FITC).

☑ The axis scales are clearly visible. Include numbers along axes only for bottom left plot of group (a 'group' is an analysis of identical markers).

☑ All plots are contour plots with outliers or pseudocolor plots.

☑ A numerical value for number of cells or percentage (with statistics) is provided.

Methodology

Sample preparation: All the samples for flow cytometry were prepared fresh, stained with

Instrument: BD Fortessa and Cytek Aurora flow cytometer was used to run flow on

Software: Flow analysis was done using FlowJo v10.8.1 (Tree Star) software.

Cell population abundance: Immune cells abundance was calculated based on their percent with

Gating strategy: Gating strategies were adopted from our own as well as other publications

☑ Tick this box to confirm that a figure exemplifying the gating strategy is provided in the Supplementary Information.

Magnetic resonance imaging

Experimental design

Design type

Design specifications

Behavioral performance measures

Imaging type(s)

Field strength

Sequence & imaging parameters

Area of acquisition

Diffusion MRI ☐ Used ☐ Not used

Preprocessing

Preprocessing software

Normalization

Normalization template

Noise and artifact removal

Volume censoring

Statistical modeling & inference

Model type and settings

Effect(s) tested

Specify type of analysis: ☐ Whole brain ☐ ROI-based ☐ Both

Statistic type for inference

(See Eklund et al. 2016)

Described in the demographics table for all respective results, also any dditional details regarding human participants will be made available through the corresponding author of the manuscript, Willa.Hsueh@osumc.edu

Correction

Models & analysis

n/a	Involved in the study
☐	☐ Functional and/or effective connectivity
☐	☐ Graph analysis
☐	☐ Multivariate modeling or predictive analysis

Functional and/or effective connectivity

Graph analysis

Multivariate modeling and predictive analysis

This checklist template is licensed under a Creative Commons Attribution 4.0 International License, which permits use, sharing, adaptation, distribution and reproduction in any medium or format, as long as you give appropriate credit to the original author(s) and the source, provide a link to the Creative Commons license, and indicate if changes were made. The images or other third party material in this article are included in the article's Creative Commons license, unless indicated otherwise in a credit line to the material. If material is not included in the article's Creative Commons license and your intended use is not permitted by statutory regulation or exceeds the permitted use, you will need to obtain permission directly from the copyright holder. To view a copy of this license, visit http://creativecommons.org/licenses/by/4.0/
